# Oral Active Carbon Quantum Dots for Diabetes

**DOI:** 10.3390/ph17101395

**Published:** 2024-10-19

**Authors:** Gamze Camlik, Besa Bilakaya, Esra Küpeli Akkol, Adrian Joshua Velaro, Siddhanshu Wasnik, Adi Muradi Muhar, Ismail Tuncer Degim, Eduardo Sobarzo-Sánchez

**Affiliations:** 1 Department of Pharmaceutical Technology, Faculty of Pharmacy, Biruni University, Istanbul 34015, Türkiye; gcamlik@biruni.edu.tr (G.C.); bbilakaya@biruni.edu.tr (B.B.); 2 Biruni University Research Center (B@MER), Biruni University, Istanbul 34015, Türkiye; 3 Department of Pharmacognosy, Faculty of Pharmacy, Gazi University, Ankara 06330, Türkiye; esrak@gazi.edu.tr; 4 Department of Surgery, Faculty of Medicine, Universitas Sumatera Utara, Medan 20155, Indonesia; ajoshuav@gmail.com (A.J.V.); adi.muradi@usu.ac.id (A.M.M.); 5 Artisan Karya Abadi Research, Medan 20155, Indonesia; 6 Department of Surgery, Dr. Djasamen Saragih Regional Public Hospital, Pematang Siantar 21121, Indonesia; 7 Faculty of Medicine, Government Medical College and Hospital, Miraj 416410, Maharashtra, India; anshuwasnik@gmail.com; 8 Instituto de Investigación y Postgrado, Facultad de Ciencias de la Salud, Universidad Central de Chile, Lord Cochrane 417, Santiago 8330507, Chile; 9 Department of Organic Chemistry, Faculty of Pharmacy, University of Santiago de Compostela, 15782 Santiago de Compostela, Spain

**Keywords:** carbon quantum dots, metformin, oral administration, type II diabetes, nanotechnology

## Abstract

Background/Objectives: Metformin (Met), an oral drug used to treat type II diabetes, is known to control blood glucose levels. Metformin carbon quantum dots (MetCQDs) were prepared to enhance the bioavailability and effectiveness of metformin. Several studies have shown that carbon quantum dots (CQDs) have attractive properties like small particle size, high penetrability, low cytotoxicity, and ease of synthesis. CQDs are made from a carbon source, namely, citric acid, and a heteroatom, such as nitrogen. The active molecule can be a carbon source or a heteroatom, as reported here. Methods: This study aims to produce MetCQDs from an active molecule. MetCQDs were successfully produced by microwave-based production methods and characterized. The effect of the MetCQDs was tested in Wistar albino rats following a Streptozocin-induced diabetic model. Results: The results show that the products have a particle size of 9.02 ± 0.04 nm, a zeta potential of −10.4 ± 0.214 mV, and a quantum yield of 15.1 ± 0.045%. Stability studies and spectrophotometric analyses were carried out and the effectiveness of MetCQDs evaluated in diabetic rats. The results show a significant reduction in blood sugar levels (34.1–51.1%) compared to the group receiving only metformin (37.1–55.3%) over a period of 30 to 360 min. Histopathological examinations of the liver tissue indicate improvement in the liver health indicators of the group treated with MetCQDs. Conclusions: Based on these results, the products have potential therapeutic advantages in diabetes management through their increased efficacy and may have reduced side effects compared to the control group.

## 1. Introduction

Metformin HCl (Met), an antidiabetic agent, is a biguanide class of drug used as the first step in pharmacological therapy for the treatment of type II diabetes. In addition, it was approved by the US Food and Drug Administration (FDA) in 1994 [1]. Met is a basic hydrophilic drug with a pKa value of 11.5. Animal and human studies have shown that it acts in the liver and inhibits gluconeogenesis by blocking mitochondrial redox shuttles [2,3,4,5]. It also increases insulin-mediated glucose use in peripheral tissue such as the liver and muscles, reduces glucose absorption in the small intestines, and lessens gluconeogenesis by reducing free fatty acid in plasma. The drug essentially activates the AMPk-activated protein kinase, which plays an important role in the anti-hyperglycemic effect [6]. Met is not a hypoglycemic drug, but an euglycemic drug, thereby reducing the potential for hypoglycemia. Several studies have shown that Met treatment is preferred for patients with type II diabetes due to its benefits, such as long-term safety and efficacy data, low risk of hypoglycemia, cardiovascular benefits, mortality benefits, additive or synergistic effects in combination therapy, low cost, and wide availability [7].

In recent years, carbon-based nanomaterials, including fullerenes, graphene, nanodiamonds, carbon nanotubes (CNTs), and carbon quantum dots (CQDs), have garnered significant attention due to their unique structural characteristics and exceptional chemical and physical properties [8]. However, the synthesis and purification of nanodiamonds present challenges, while other nanomaterials, such as graphene, fullerenes, and CNTs, suffer from poor water solubility and lack strong visible-region fluorescence [9]. As mentioned in the literature, CQDs can be produced with simple and easy microwave-assisted technology at a low temperature and power (100–200 W). The production is simple: if the color changes to dark brown, it can be accepted as an indication of the presence of graphene quantum dots. A clear solution obtained after synthesis can indicate the presence of CQDs [10]. Although semiconductor quantum dots (QDs) show good fluorescent properties, these materials are toxic and are obtained from heavy metals. Consequently, their biological application in biosensors, bioimaging, and drug delivery are limited [11,12]. To overcome these limitations, CQDs have been reported to have attractive properties, such as high photostability, low cytotoxicity, ease of synthesis, good water solubility, a high photo-response, easy surface functionalization, good catalysis, and tunable excitation–emission [13,14].

According to previous studies, CQDs can be used in imaging as well as in drug delivery systems due to their nature. The drug delivery system protects against degradation (enzymatic) as well as reduces the number of doses required and their side effects, leading to increased effectiveness and bioavailability [15].

In this study, MetCQD formulations were produced and tested for better results. The formulations were prepared using citric acid as a carbon source and Met was used for dual-action purposes as a heteroatom and an active molecule. The mitochondrial accumulation properties of Met were also considered. The initial hypothesis considers the action of MetCQDs on mitochondria, which is essential for the treatment of diabetes and many other diseases related to mitochondrial dysfunction.

## 2. Results

### 2.1. The One-Step Production of MetCQDs

**Figure 1:**
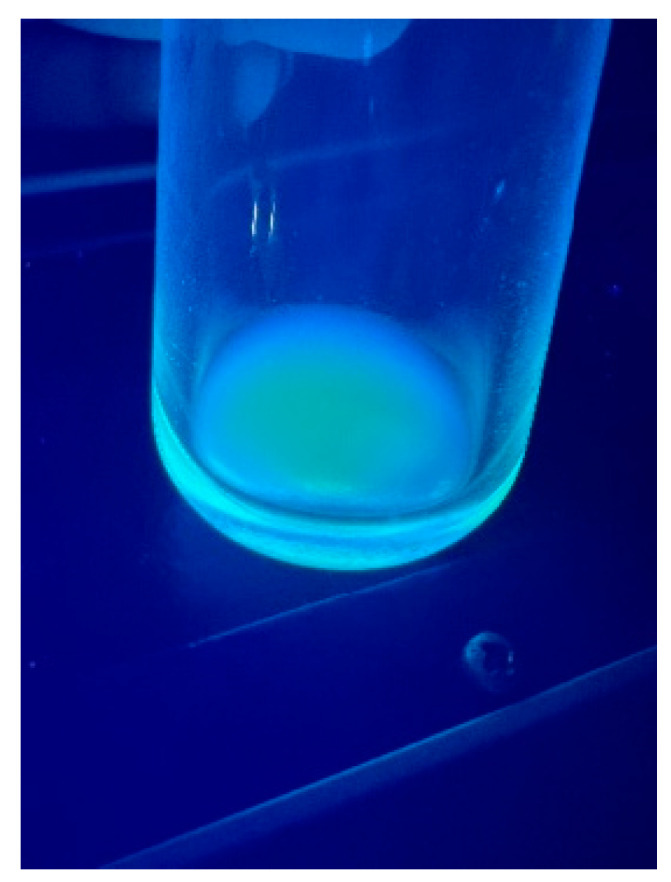
The physical appearance of MetCQDs under UV light (365 nm).

MetCQDs were successfully synthesized and the process proved to be straightforward and easily applicable. A total of 0.2 g of citric acid monohydrate was used as a carbon source, with 0.5 g of Met used as both a heteroatom and an active pharmaceutical ingredient. The method was found to be cost-effective and rapid. A blue-green fluorescent emission was observed under UV light (365 nm) after the reaction (Figure 1). This served as a rapid initial confirmation and the presence of the blue emission showed the completion of a successful reaction, as failure would result in the absence of this emission.

### 2.2. Characterization of MetCQDs

#### 2.2.1. Physical Appearance, Particle Size and Distribution, and Zeta Potential

The analysis of MetCQDs involved an evaluation of their particle size (PS), distribution, and zeta potential (ZP) using the Lite Sizer 500 from Anton Paar (Anton Paar, St. Albans Hertfordshire, AL4 0LA, UK) (Table 1).

**Figure 2:**
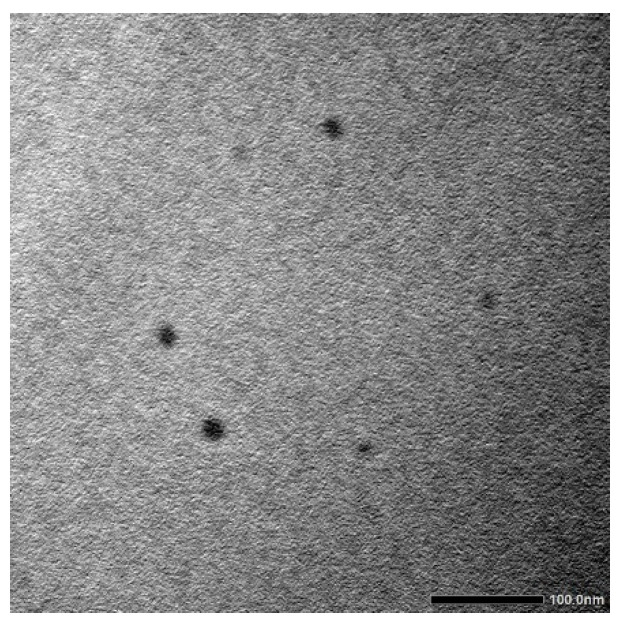
Physical appearance of MetCQDs under TEM.

The prepared MetCQDs were diluted with water then imaged using a TEM (Figure 2). It was observed that the MetCQDs were of a very small size, measuring less than 10 nm.

#### 2.2.2. Fluorescence

The MetCQDs were used to examine fluorescence, showing successful preparation through the observation of vivid blue emissions. Exciting this fluorescence with 365 nm of light verified the optimal emissions, as confirmed by a spectrofluorometer (RF-6000 fluorescence spectrophotometer, Shimadzu, Kyoto, Japan). However, using a longer wavelength of UV light (420 nm) led to a shift in the emissions toward the red spectrum. Factors such as the selection of the carbon source and pH prove to have an influence on both surface property and doped atom selection. The prepared MetCQDs appeared as a blue-green fluorescence under UV light.

#### 2.2.3. Quantum Yield

Quinine sulfate solution served as the established standard for comparison. The emissions obtained from the MetCQDs exhibited a satisfactory intensity leading to a calculated quantum yield deemed sufficiently high. Graphs plotting the intensity against the wavelength for both the quinine sulfate and the MetCQDs were generated. In the experiment, the slope of the area under the curve (AUC)–intensity relationship for the quinine sulfate measured 895.72, while the CQDs registered at 720.34 with an AUC value of 0.804. Consequently, the quantum yield was determined to be 80.3%.

#### 2.2.4. Stability

The characterization parameters (particle size and distribution and zeta potential) of the prepared formulations were kept at different temperatures (−20 °C, 5 °C, 25 °C, and 40 °C) during stability testing and the shelf life was calculated at more than 2 years. The relative humidity values of the formulations were 60% and 75% at 25 °C and 40 °C, respectively. There was no significant change in the results obtained.

### 2.3. Assay

The amount of Met was successfully determined by the spectrophotometric method and was found to be linear, repeatable, and stable. The dose given to the animals was kept constant by controlling the quantification.

#### 2.3.1. In Vivo Experiments

Animals were grouped, and the experiment proceeded according to the protocol in the Materials and Methods Section. No animal died and no animal was excluded from the experiment.

#### 2.3.2. Determination of Blood Glucose Levels

The animals’ blood glucose levels were measured successfully by following the protocol specified in the Materials and Methods Section. There were no problems related to the measurement and method used.

#### 2.3.3. Effects on Normal and Glucose-Loaded Rats (NG-OGTT)

**Figure 3:**
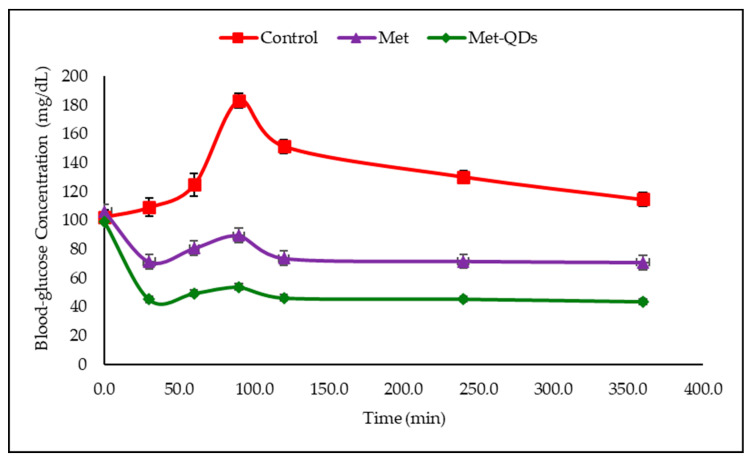
Blood glucose concentration graph in healthy animals.

A significant decrease in blood glucose level was achieved after metformin administration. The test material was found to show stronger hypoglycemic activity compared to metformin (Table 2 and Figure 3).

#### 2.3.4. Acute Antidiabetic Effect

**Figure 4:**
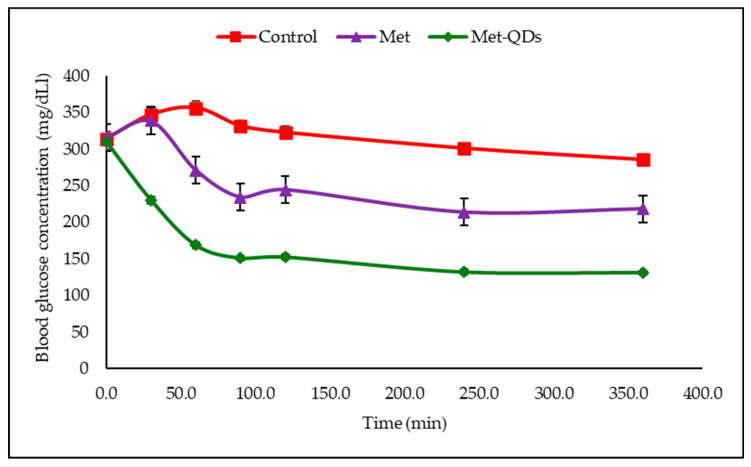
Blood glucose concentration graph in diabetic animals.

A total of 60 min after oral administration, a 23.9–31.1% decrease in blood glucose was observed in the metformin-administered group and a 28–35.2% decrease in the test-material-applied group (Table 3 and Figure 4).

### 2.4. Histopathological Examinations

In this experimental study, the pathological results from the pancreata and livers of rats with type II diabetes and the changes observed as a result of the application of the test samples were examined, and the macroscopic and microscopic pathological results detected given below.

#### 2.4.1. Macroscopic Observations

The pancreata and livers of the rats, necropsied after euthanasia, were examined to determine whether there were any macroscopic lesions. A remarkable finding was observed.

#### 2.4.2. Histopathological Observations

##### Histopathological Results of the Pancreatic Tissue

**Figure 5:**
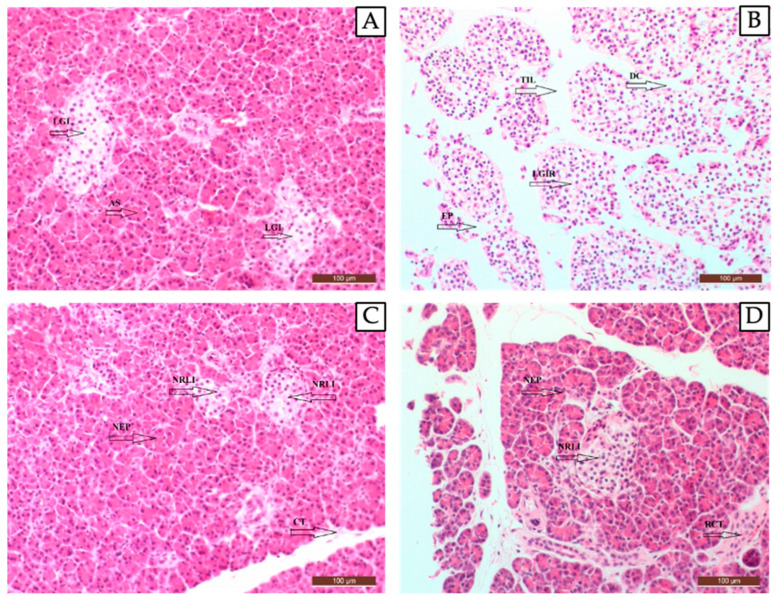
A histopathologic view of pancreatic tissue in the control group (healthy animals) (**A**), diabetic control group (**B**), metformin-administered group (**C**), and MetCQD-administered group (**D**). **AS**: acinus (exocrine pancreas), **CT:** normal connective tissue structure, **DC:** destruction of the connective tissue that separates the pancreatic lobules from each other, **EP:** exocrine pancreatic structure degeneration, **LGI:** Langerhans islet (pancreatic islet cells, endocrine pancreas), **LGIR:** Langerhans islets ruptured, **NEP:** new exocrine pancreatic structure, **NRLI:** new regeneration of the Langerhans islet, and **RCT:** regeneration of the connective tissue structure that separates the pancreatic lobules from each other. 40X.

The pancreatic tissue of rats in the control group exhibited a normal histological structure (Figure 5A). Rats in the STZ-induced diabetic group showed degenerative and necrotic changes in the endocrine portion of the pancreas, identified by the atrophic appearance of the islets of Langerhans in the endocrine pancreata due to degenerative and necrotic changes (Figure 5B). In the STZ + Metformin group, it was observed that the necrotic and degenerative changes decreased compared to the other experimental groups; a new pancreatic exocrine structure formed and regeneration occurred in the islets of Langerhans containing the endocrine portion of the pancreas (Figure 5C). In the STZ + Test material group, regeneration was observed in the exocrine gland epithelium, in the islets of Langerhans containing the endocrine portion of the pancreas, and in the connective tissue separating the pancreatic lobules (Figure 5D).

##### Histopathological Results of the Liver Tissue

**Figure 6:**
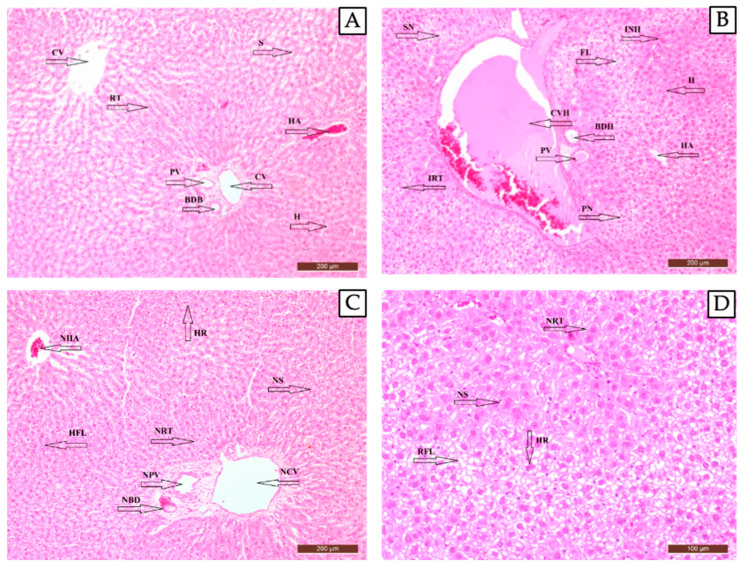
A histopathologic view of liver in the control group (healthy animals) (**A**), diabetic control group (**B**), STZ + Metformin-administered group (**C**), and STZ + MetCQD-administered group (**D**). 200X. **BDB:** bile duct branch, **BDH:** bile duct hyperplasia, **CV**: central vein, **CVH:** central vein hyperplasia, **FL:** fatty liver, **H:** hepatocytes, **HA:** hepatic artery, **HFL:** healing fatty liver, **HR:** hepatocytes regeneration (binucleated hepatocytes), **INH:** increase in number of large hepatocytes, **IRT:** irregular trabeculae, **NBD:** normal bile duct branch, **NCV:** normal central vein, **NHA:** normal hepatic artery, **NPV:** normal portal vein, **NRT:** new regular trabeculae formation, **NS:** new sinusoid structure formation, **PN:** periacinar necrosis, **PV:** portal vein, **RFL:** reduction in fatty liver, **RT:** regular trabeculae, **S:** sinusoid, and **SN:** sinusoid narrowing.

The liver tissue from rats in the control group was observed to have a normal histological structure (Figure 6A). In the diabetic control group, fatty degeneration, widespread hepatocyte enlargement, irregular trabecular structure, periacinar necrosis, and sinusoidal constriction were observed (Figure 6B). In the STZ + Metformin group, improvement in liver fat content, regeneration in hepatocytes, regeneration in the bile duct, improvement in the sinusoidal structure, a regular trabecular network, and a normal structure of the hepatic artery, central vein, and portal vein were observed (Figure 6C). In the STZ + MetCQDs group, regeneration, a regular trabecular network, and decreased liver fat content were identified due to increased mitotic activity in the hepatocytes (Figure 6D). 

## 3. Discussion

CQDs, known as excellent fluorescent nanomaterials, have a particle size of less than 10 nm, representing no optical vibration, a high fluorescence stability, a continuous and tunable emission wavelength, a wide excitation spectrum, good biocompatibility, and low toxicity. Due to these properties, they are widely used in biological systems for detection, in vivo and in vitro imaging, drug delivery, and diagnostic medicine [16,17]. Additionally, while they can be prepared from a carbon source, it is known that the active ingredient itself can be used as a carbon source.

The CQDs in this study were obtained from the parent active substance metformin HCl. The particle size and polydispersity index, zeta potential, and quantum yield were found to be 9.02 ± 0.04 nm, 19.2% ± 0.102, 15.1 ± 0.045 mV, and 80.3%, respectively. In addition, TEM images showed homogeneous dispersion and no aggregation, proven by the PDI. This was also reported in the literature; homogeneous dispersion was obtained when the PDI was less than 20% [18]. This obtained value showed that the prepared formulations were homogeneous.

Met is an oral antidiabetic agent used to treat type II diabetes and is used as a first-line treatment. Metformin is shown to improve glycemic control, reduce hyperglycemia, and prevent the occurrence of diabetes-related complications [19]. Additionally, its mechanism of action can include the inhibition of glucose production in the liver and increased insulin sensitivity. The study proposed in this article suggests that it can increase the effectiveness of metformin in the treatment of type II diabetes, with potential benefits for its management, particularly through the use of nanotechnology [20].

Characterization parameters such as particle size and distribution and zeta potential of the prepared MetCQD formulations were continuously maintained at temperatures of −20 °C, 5 °C, 25 °C, and 40 °C during stability testing and the shelf life was calculated at more than 2 years. The relative humidity values of the formulations were identified as 60% and 75% for 25 °C and 40 °C, respectively. This indicates there was no significant change for all formulations.

The active substance Met was successfully measured at 232 nm and the method was determined to be linear, specific, selective, repeatable, and stable.

Met was slowly and incompletely absorbed after oral administration and resulted in some significant side effects when administered orally [2]. The most serious complication was metformin-associated lactic acidosis (MALA), with a mortality rate ranging from 25% to 50%. This term, classically, included any metformin user with increased lactate. In 2017, Lalau et al. described a series of clinical conditions such as metformin-induced lactic acidosis (MILA) and metformin-unrelated lactic acidosis (MULA). This occurred when high metformin serum levels were the underlying cause of patient death [21]. Therefore, the dose must be lowered to avoid unwanted side effects. The MetCQDs were prepared to increase their bioavailability at lower doses and to reduce their side effects. It was observed that although the same dose was administered, the effect with the MetCQDs was greater; to have the same effect, the dose should be minimized. In this way, the side effects could be lessened.

In this study, Wistar albino rats were used to test the effect of MetCQDs. In an environment where the temperature, relative humidity, and light/dark cycles were controlled, the rats were fed with the standard feed and clean drinking water. In the experiment, the blood glucose levels of the rats were measured and then divided into different groups, namely: Group I (control), Group II (STZ, diabetic control), Group III (STZ + Met), and Group IV (STZ + test sample (MetCQD)). An oral glucose tolerance test was performed and fasting blood sugar levels were determined.

A significant reduction in blood glucose levels after Met administration was observed (Table 1 and Figure 3) when healthy animals were used. However, a stronger hypoglycemic effect was observed in rats administered with MetCQDs than with Met alone. The same, stronger effect was observed with the MetCQDs when diabetic animals were subjected to the test (Table 2 and Figure 4).

At the end of the experiment, the rats were euthanized under anesthesia and the pancreas and liver tissue were prepared and evaluated for histopathological examination. As a result of these evaluations, it was determined that the liver tissue of the rats in the control group (Group I) had a normal histological structure (Figure 5A). In the diabetic control group (Group II), fatty liver, hepatocyte expansion, irregular trabecular structure, periacinar necrosis, and sinusoid narrowing were observed (Figure 5B). In the STZ + Metformin group (Group III), improvement in liver steatosis, hepatocyte regeneration, improvement in the bile duct and sinusoid structure, a regular trabecular network structure, and normal hepatic artery and portal vein structures were observed (Figure 5C). In the STZ + Test Material (MetCQDs) group (Group IV), increased mitotic activity in hepatocytes, along with regeneration, a regular trabecular network, and decreased fatty liver were observed (Figure 5D).

Liver tissue and cells were found to be normal in the control group (Figure 6A), whereas some fatty degeneration, enlarged hepatocytes with irregular trabecular structures, some necrosis, and sinusoidal constrictions were observed in the diabetic animals (Figure 6B). This was found to be proof of the STZ effect in the diabetic animal model. Met was found to be effective in improving liver fat content and some regeneration was also observed. Some regeneration in the bile duct, improvement in the sinusoidal structure, a regular trabecular network, and normal structures of the hepatic artery, central vein, and portal vein were observed (Figure 6C). However, even more regeneration, a regular trabecular network, accepted to be an indication of good regeneration, and decreased liver fat content were identified, and increased mitotic activity in hepatocytes was observed in the MetCQD-administered group of diabetic animals (Figure 6D). All these were attributed to the effects of CQDs previously described [12].

Literature reports demonstrate the ability of CQDs to traverse multiple membrane barriers [22]. Many reports also show that positively charged quantum dots can penetrate cell membranes much faster. MetCQDs represent a negative surface charge and penetrate biological membranes, as well. This shows that the penetration mechanism is more complex than just a charge; some other mechanism may be involved. The cellular uptake and subsequent cellular toxicity may also differ from the type of quantum dot. Furthermore, positively charged quantum dots have been shown to exhibit enhanced cellular membrane permeability. A 2020 study reported the unintentional formation of CQDs while cooking salmon in an oven at 200 °C. The same research indicated no histopathological effects in rats at a dose of 2 g/kg [23]. A 2015 study investigating pancreatic targeting with quantum dots observed reduced blood glucose levels three to four weeks post-administration [24]. Observations of enhanced metabolic activity in the mitochondria were reported. This may explain why some regeneration was observed in the tissue of the diabetic animals.

Cases of diabetes and related diseases have risen and the number of patients has grown exponentially around the world. Impaired glucose tolerance could result in high blood glucose levels, which severely endanger people. Therefore, controlling blood glucose levels is essential to therapy, and there are several drugs available to reduce blood glucose levels on the market. Biguanides or sulfonylureas can be used to successfully treat high blood glucose levels, but have some side effects [25,26]. The CQDs presented here appear to be a good alternative and are a subclass of carbon material used in various biological applications because of their low toxicity and good biocompatibility [15,27,28]. A carbon atom can have four bonds with other atoms; hence, it can be a chiral atom. Chirality is a common phenomenon and plays a critical role in physiological activities [29,30]. The CQDs in this study were made of carbon and nitrogen atoms and used as heteroatoms. Although it is not generally known and there are limited sources available in the literature, MetCQDs are more effective at reducing blood glucose levels, as mentioned here.

In studies conducted with CQDs, it was found that CQDs increased metabolic activity by affecting the energy pathways of the cells and by going to the mitochondria of the cells; therefore, they had a hypoglycemic effect [12].

## 4. Materials and Methods

### 4.1. Materials

Citric acid monohydrate and polyethylene glycol (PEG 3350) were purchased from Sigma Aldrich (St. Louis, MO, USA). The metformin HCl was donated by SANOVEL, and a microwave reactor (Anton Paar Microwave 300, Anton Paar, St. Albans Hertfordshire, AL4 0LA, UK), was used for the synthesis of the MetCQDs. Additionally, particle size, size distribution, and zeta potential were determined using an Anton Paar LiteSizer 500. The optical properties of the MetCQDs were characterized using a spectrofluorometer (Model 229129, Agilent Technologies, Santa Clara, CA, USA). For use in this experimental in vivo study, Wistar albino rats weighing 200–250 g were obtained from the Kobay Animal Study Center (Ankara, Turkey). The experiments were conducted with the approval of the ethics committee of the Ankara University Faculty of Pharmacy in the Department of Pharmacology (Local Ethics Committee for Animal Experiments, decision no. 044). This study adhered to the Animal Study Reporting In Vivo Experiments (ARRIVE) guidelines. Fasting blood glucose levels were measured using a Bayer Glucometer Elite. Streptozocin (St. Louis, MO, USA) (dissolved in 0.1 M of citrate buffer with a pH of 4.50) was used to induce diabetes in the animals, and sodium carboxymethylcellulose (Sigma-Aldrich, St. Louis, MO, USA) was administered to the rats in the STZ groups. Furthermore, a formalin solution was used for the macroscopic and histopathological examinations. An automatic tracking device (SHANDON Citadel 1000, Brentwood, TN 37027, United States) and microtome (Reichert rotary, Buffalo City, NY 14043, United States) devices were used for the histopathology examinations and the hematoxylin–eosin method was used for section staining. Microscopic examinations were performed with an Olympus BX51 (Olympus, Tokyo, Japan) (DP72 camera attachment) light microscope.

The SPSS 20.0 program was used for the statistical evaluation, and the Kruskal–Wallis and Mann–Whitney U tests were used for the histopathological examination.

### 4.2. Methods

#### 4.2.1. The One-Step Production of MetCQDs

The effective conditions and parameters impacted the dimensions and surface properties of the MetCQDs and lead to the optimization of the production method. Additionally, to create the MetCQDs, 0.2 g of citric acid monohydrate served as a carbon source, while 0.5 g of Met acted as the active drug source. All chemicals were dissolved in 1 mL of distilled water and transferred to the microwave reactor. The heating program adhered to the following steps:

- Heat up to 160 °C for 5 min;
- Maintain a temperature of 160 °C for 20 min;
- Cool gradually to reach room temperature.
- Finally, the MetCQDs were successfully created and checked under UV light (365 nm) for rapid confirmation. After the reaction, surface modification was achieved by adding 0.02 g of PEG 3350.

#### 4.2.2. Characterization of the MetCQDs

##### Physical Appearance, Particle Size and Distribution, and Zeta Potential

Characterization studies were conducted on the MetCQDs and a transmission electron microscope (TEM, Carl Zeiss EM 900, Jena, Germany) was used to ascertain their particle shape and morphological traits. Particle size and distribution were determined using an Anton Paar LiteSizer 500. All measurements were performed with 6 replicates for size, distribution, and zeta potential determination.

##### Fluorescence

A spectrofluorometer was used to determine the optical characteristics of the MetCQDs. Excitation of the MetCQDs allowed for the recording of their emissions while their response under UV light was observed and documented.

##### Quantum Yield

The calculation method used was adapted from a previously conducted study in the literature [11,14]. First, the absorbance of quinine sulfate in a sulfuric acid solution was gauged at 350 nm. Subsequently, the complete fluorescence spectrum, depicting fluorescent intensity across various wavelengths, was generated for the solution. Dilution steps were undertaken in increments of 0%, 20%, 40%, 60%, 80%, and 100% of the original solution, with fluorescence spectra plotted for each. The areas beneath these curves (AUC) were calculated using the trapezoidal method and the resultant AUC values were correlated with the intensity to determine the slope value. This entire procedure was replicated for the MetCQD solution, yielding a comparable fluorescence spectrum. Quantum yield was calculated as the following:

Quantum yield = {m(test)/m (quinine sulphate)} * A,

where A = (refractive index-water/refractive index-0.1 M sulfuric acid) = 0.99939985

[Refractive index-water = 1.3325, refractive index-0.1 M sulfuric acid = 1.3333]

and m is the slope of the AUC-intensity graphic.

#### 4.2.3. Assay

A Met standard solution was prepared and a UV spectrophotometer was used. The maximum wavelength (λ max) was 232 nm and the calibration curve was obtained using standard solutions. Additionally, the curve was found to be linear and the method reproducible. The other validation parameters were suitable, such as repeatability, specificity, stability, etc. [31].

##### Stability

Stability tests were performed to determine chemical and physical stability and the shelf life of the MetCQDs.

##### The In Vivo Experiments

Wistar albino rats weighing 200–250 g were obtained from the Guinea pig laboratory and housed in a room at a temperature of 22 ± 2 °C with approximately 60% relative humidity and a 12 h light/dark cycle. The experimental animals were fed with standard rat chow and their drinking water bottles were changed daily. Animals were randomly selected and groupings of 7 animals in each experimental group were created.

##### The Determination of Blood Glucose Levels

The blood glucose concentration of each rat was measured from a fasting state. Blood samples were collected from their tails and the Glucometer Elite commercial test (Bayer), based on the glucose oxidase method, was used to determine their blood glucose levels.

##### The Effects on Normal and Glucose-Loaded Rats (NG-OGTT)

Animals that received the Met solution or the MetCQCs were administered orally. Blood glucose levels were measured at the beginning and after 30 and 60 min, followed by an oral glucose tolerance test. The rats were orally loaded with glucose at a dose of 2 g/kg and their blood glucose levels determined at the 90th, 120th, 240th, and 360th min [32,33].

##### The Diabetes Model

The rats were made diabetic by administering a streptozotocin solution (STZ, 60 mg/kg, i.p.) (St. Louis, MO, USA) dissolved in 0.1 M of a citrate buffer solution with a pH of 4.50. The control group was given a citrate buffer solution that did not contain STZ. After 3 weeks of streptozotocin injections, a drop of blood was collected from the tail vein and the fasting blood glucose level was measured using a blood glucose monitoring system (One Touch^®^ UltraMini^®^, Life Scan, Inc., Milpitas, CA, USA). Rats with a blood glucose concentration level of ≥300 mg/dL were considered diabetic [34,35,36].

Groups:

**Group I:** control group; the group without any experimental procedure (*n = 7*).

**Group II (STZ, Diabetic control):** animals in which diabetes was induced with STZ (60 mg/kg, single dose i.p., 1 mL) (*n = 7*).

**Group III (STZ + Met):** diabetic animals given a pure metformin solution (60 mg/kg, single dose i.p., 1 mL, and Met 250 mg/kg; per os) (*n = 7*).

**Group IV (STZ + MetCQDs):** diabetic animals given new formulations of Metformin HCl (60 mg/kg, single dose i.p., 1 mL, and MetCQDs 250 mg/kg; per os) (*n = 7*).

The administered dose of Met was calculated relative to the clinically relevant human dose based on body surface area.

Metformin dissolved in 0.5% sodium carboxymethylcellulose (250 mg/kg/day, Sigma-Aldrich, St. Louis, MO, USA) was administered via gastric gavage. Rats in the STZ groups were administered 0.5% sodium carboxymethylcellulose.

The acute antidiabetic effect of the test samples was determined from a fasting state and the blood sugar levels were measured at 30, 60, 90, 120, 240, and 360 min after the application of Met and the MetCQDs. At the end of the experiment, the rats were euthanized with a high dose of anesthesia. Pancreas and liver tissue were taken and placed in a 5% formalin solution until use.

##### Histopathological Examination

Tissue pieces prepared in a block size 0.5 cm thick were passed through the alcohol and xylol series for dehydration, clearing, and paraffinization processes with an automatic tracking device (SHANDON Citadel 1000). A total of 5 micron-thick sections were taken from the prepared paraffin blocks using a microtome (Reichart rotary), and all sections were stained using the hematoxylin–eosin staining method. Microscopic examination of the preparations were performed with an Olympus BX51 (DP72 camera attachment) light microscope. Histopathological results were observed in sections from 10 random areas examined under the light microscope and were evaluated according to the atrophic appearance of the islets of Langerhans in the pancreas, normal (−), mild (+), moderate (++), and severe (+++); in the liver, (−), mild (+), moderate (++), and severe (++); and in terms of necrotic and degenerative change, evaluated as (+).

##### Statistical Analysis

The SPSS 20.0 program was used for statistical evaluation. In histopathological examinations, the difference between the groups in the data regarding the atrophic appearance of the islets of Langerhans in the pancreas and the necrotic-degenerative changes in the liver was determined by the Kruskal–Wallis test. The detection of the groups creating the difference was determined by the Mann–Whitney U test (a *p* < 0.05 value is considered statistically significant).

## 5. Conclusions

In conclusion, MetCQDs were prepared using citric acid as a carbon source and Met was used for dual-action purposes as a heteroatom and an active molecule for the first time. The MetCQDs formulations were successfully produced and exhibited a hypoglycemic effect; the liver fat was also found to have decreased. This study concludes that the preparation of quantum dot formulations of active substances can open the door to new treatment alternatives that use drug molecules in both roles. Finally, further studies are necessary to determine the possible long-term effects.

## Figures and Tables

**Table 1:** Particle size, zeta potential, and polydispersity index of prepared MetCQDs.

Formulation	Particle Size (nm)	Zeta Potential (mV)	Polydispersity Index (%) (PDI %)
MetCQDs	9.02 ± 0.04	−10.4 ± 0.214	15.1 ± 0.045

**Table 2:** Acute effect of test samples on blood glucose levels in normal and glucose-loaded hyperglycemic (NG-OGTT) rats.

Group	Blood Glucose Concentration ± S.E.M. (Inhibition %)						
	0 min	30 min	60 min (+Glucose)	90 min	120 min	240 min	360 min
Control	102.5 ± 4.8	109.2 ± 6.3	124.7 ± 6.1	183.0 ± 5.2	151.3 ± 4.8	130.2 ± 4.4	114.6 ± 4.9
Metformin	106.2 ± 5.7	71.2 ± 3.9 *	80.6 ± 3.1 **	89.4 ± 3.6 **	73.6 ± 3.2 **	71.6 ± 3.5 **	70.8 ± 4.2 *
MetCQD	99.3 ± 4.1	45.1 ± 3.7 **	49.4 ± 3.2 **	53.8 ± 3.3 **	46.3 ± 3.1 **	45.5 ± 3.2 **	43.7 ± 3.0 **

*: *p* < 0.01; **: *p* < 0.001; S.E.M.: standard error of the mean.

**Table 3:** Effect of test samples on blood glucose levels in diabetic rats with STZ.

Groups	Blood Glucose Concentration ± S.E.M. (Inhibition %)						
	0 min	30 min	60 min	90 min	120 min	240 min	360 min
**Diabetic Control**	314.3 ± 9.9	347.1 ± 10.8	356.3 ± 9.1	331.5 ± 8.2	322.7 ± 8.4	310.1 ± 6.3	285.5 ± 4.6
**STZ + Metformin**	315.9 ± 8.5	338.6 ± 8.2	271.2 ± 6.3 *	234.2 ± 5.1 *	244.5 ± 5.0 *	213.7 ± 5.1 **	218.2 ± 4.9
**STZ + MetCQDs**	309.7 ± 10.4	229.9 ± 4.9	168.6 ± 4.1 **	150.9 ± 3.3 **	152.5 ± 3.5 **	132.1 ± 3.4 **	131.3 ± 2.2 **

*: *p* < 0.05; **: *p* < 0.01; S.E.M.: standard error of the mean.

## Data Availability

Dataset available on request from the authors.
